# Reciprocal regulation of oxidative stress and mitochondrial fission augments parvalbumin downregulation through CDK5-DRP1- and GPx1-NF-κB signaling pathways

**DOI:** 10.1038/s41419-024-07050-5

**Published:** 2024-09-30

**Authors:** Su Hyeon Wang, Duk-Shin Lee, Tae-Hyun Kim, Ji-Eun Kim, Tae-Cheon Kang

**Affiliations:** Department of Anatomy and Neurobiology, Institute of Epilepsy Research, College of Medicine, Hallym University, Chuncheon, 24252 South Korea

**Keywords:** Epilepsy, Cell death in the nervous system

## Abstract

Loss of parvalbumin (PV) expressing neurons (PV neurons) is relevant to the underlying mechanisms of the pathogenesis of neurological and psychiatric diseases associated with the dysregulation of neuronal excitatory networks and brain metabolism. Although PV modulates mitochondrial morphology, volume and dynamics, it is largely unknown whether mitochondrial dynamics affect PV expression and what the molecular events are responsible for PV neuronal degeneration. In the present study, L-buthionine sulfoximine (BSO, an inhibitor of glutathione synthesis) did not degenerate PV neurons under physiological condition. However, BSO-induced oxidative stress decreased PV expression and facilitated cyclin-dependent kinase 5 (CDK5) tyrosine (Y) 15 phosphorylation, dynamin-related protein 1 (DRP1)-mediated mitochondrial fission and glutathione peroxidase-1 (GPx1) downregulation in PV neurons. Co-treatment of roscovitine (a CDK5 inhibitor) or mitochondrial division inhibitor-1 (Mdivi-1, an inhibitor of mitochondrial fission) attenuated BSO-induced PV downregulation. WY14643 (an inducer of mitochondrial fission) reduced PV expression without affecting CDK5 Y15 phosphorylation. Following status epilepticus (SE), CDK5 Y15 phosphorylation and mitochondrial fission were augmented in PV neurons. These were accompanied by reduced GPx1-mediated inhibition of NF-κB p65 serine (S) 536 phosphorylation. N-acetylcysteine (NAC), roscovitine and Mdivi-1 ameliorated SE-induced PV neuronal degeneration by mitigating CDK5 Y15 hyperphosphorylation, aberrant mitochondrial fragmentation and reduced GPx1-mediated NF-κB inhibition. Furthermore, SN50 (a NF-κB inhibitor) alleviated SE-induced PV neuronal degeneration, independent of dysregulation of mitochondrial fission, CDK5 hyperactivation and GPx1 downregulation. These findings provide an evidence that oxidative stress may activate CDK5-DRP1- and GPx1-NF-κB-mediated signaling pathways, which would be possible therapeutic targets for preservation of PV neurons in various diseases.

## Introduction

Parvalbumin (PV) is one of the Ca^2+^-binding proteins, which plays as a buffer to sequester intracellular Ca^2+^ [1]. In the brain, PV expresses in fast-spiking γ-aminobutyric acid (GABA)-ergic inhibitory interneurons characterized by high-frequency spikes of action potentials (high-frequency firing rate) with large afterhyperpolarization [2, 3]. PV expressing neurons (PV neurons) mainly receive inputs from excitatory neurons (more than 90%) and modulate the activity of neuronal network activities responsible for sustaining precise neuronal excitation/inhibition balance in feedback and feedforward manners [2–5]. To maintain properly their fast-spiking activities, PV neurons require high bioenergetics [2, 6, 7]. This high energy consumption in PV neurons consequently generates higher reactive oxygen species (ROS) levels during mitochondrial respiration at physiological level of neuronal activity [6, 8–10]. Therefore, this high reliability on mitochondrial energy metabolism makes PV neurons be extremely vulnerable to metabolic and oxidative stresses, which plays an important role in the underlying mechanisms of the pathogenesis of neurological and psychiatric diseases associated with the dysregulation of neuronal excitatory networks and brain metabolism [5]. Indeed, status epilepticus (SE, a prolonged seizure activity with 5 min or more) reduces PV expression through P/Q-type voltage-gated Ca^2+^ channel (VGCC)-mediated Ca^2+^ influx and subsequently leads to rapid PV neuronal damage in the hilus of the dentate gyrus, which cause impaired GABA release from PV neurons [11–17].

Of note, PV also modulates changes in mitochondrial morphology, volume and dynamics: PV overexpression reduces mitochondrial volume and mitochondrial fusion rates, while PV ablation increases mitochondria volume and augments ROS production in PV neurons [18–20]. Considering these inverse regulations of PV expression and mitochondrial properties, it is presumable that SE-induced PV downregulation [11–15] would evoke mitochondrial hyperfusion (elongation). However, SE-induced PV neuron degeneration is relevant to excessive mitochondrial fission by cyclin-dependent kinase 5 (CDK5)-mediated dynamin-related protein 1 (DRP1) hyperactivation [21, 22]. Since mitochondrial homeostasis is an essential housekeeping function to maintain neuronal viability and excessive mitochondrial fission provokes apoptosis [19, 23], it is noteworthy elucidating whether PV downregulation is a cause of aberrant mitochondrial dynamics in response to SE in PV neurons or a secondary aspect of PV neuronal degeneration.

On the other hand, oxidative stress is involved in PV downregulation and/or PV neuronal degeneration. Indeed, an endogenous antioxidant glutathione (GSH) and its precursor N-acetylcysteine (NAC) attenuate PV neuronal loss in various neurological models [24–26]. We have also reported that nicotinamide adenine dinucleotide phosphate oxidase (NADPH oxidase) inhibition attenuates PV cell loss by abolishing PV downregulation and excessive mitochondrial fragmentation following SE [21]. Therefore, it is likely that oxidative stress may be an upstream signaling pathway in PV neuronal loss induced by SE. However, it is unknown how oxidative stress inversely regulate PV downregulation and aberrant mitochondrial dynamics during PV neuronal degeneration, and what signals intervene in this process following SE. In order to explicate these questions, we clarified (1) whether mitochondrial dynamics affect PV expression under physiological and post-SE conditions, and (2) what the molecular events are responsible for SE-induced PV neuronal degeneration.

Here, we demonstrate that under physiological condition L-buthionine sulfoximine (BSO)-induced inhibition of GSH biosynthesis facilitated CDK5-mediated mitochondrial fission in PV neurons, which subsequently diminished PV and glutathione peroxidase-1 (GPx1) expressions. In addition, induction of mitochondrial fission resulted in reduced PV expression without affecting CDK5 tyrosine (Y) 15 phosphorylation. Following SE, however, mitochondrial fragmentation augmented CDK5 Y15 phosphorylation as well as PV downregulation. Furthermore, SE disrupted GPx1-mediated nuclear factor-κB (NF-κB) inhibition that decreased PV expression independent of altered mitochondrial dynamics. These findings indicate that both CDK5-DRP1-mediated mitochondrial fission and impaired GPx1-mediated NF-κB inhibition may diminish PV expression, which may lead to PV neuronal degeneration following SE.

## Results

### BSO-induced oxidative stress facilitates CDK5-DRP1-mediated mitochondrial fission and downregulates PV and GPx1 expressions in PV neurons under physiological condition

**Fig. 1:**
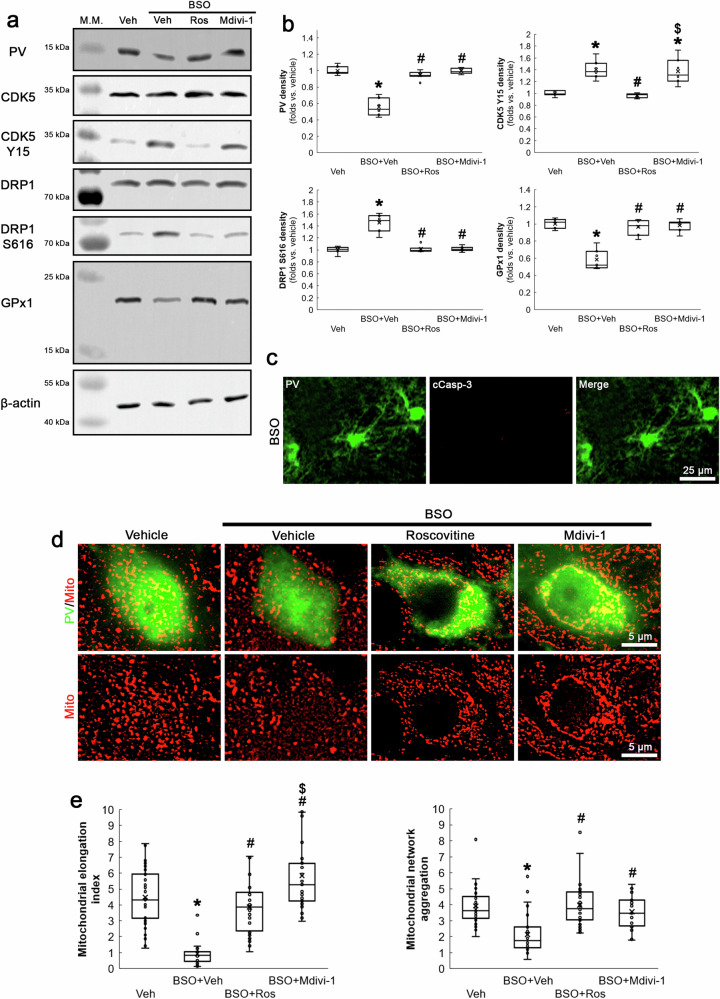
Effects of roscovitine and Mdivi-1 on BSO-induced PV downregulation, CDK5 and DRP1 hyperactivations, reduced GPx1 expression and mitochondrial fission in the rat dentate gyrus under physiological condition. BSO decreases PV and GPx1 expressions, while it increases CDK5 Y15 and DRP1 S616 phosphorylations accompanied by mitochondrial fragmentation without inducing cleaved caspase-3 (cCasp-3) in PV neurons (PV cell degeneration). Roscovitine (Ros) abrogates these phenomena. Mdivi-1 attenuates BSO-induced PV downregulation, DRP1 S616 phosphorylation, reduced GPx1 expression and mitochondrial fission without affecting CDK5 Y15 phosphorylation. **a** Representative Western blot images of PV, CDK5 Y15, DRP1 S616 and GPx1 levels in the dentate gyrus. **b** Quantification of PV, CDK5 Y15, DRP1 S616 and GPx1 levels based on Western blot data (^*,#,$^*p* < 0.05 vs. vehicle, BSO and BSO + Ros-treated animals, *n* = 7, respectively). **c** Representative images of cCasp-3 expression in PV neurons. **d** Representative images of mitochondria in PV neurons. **e** Quantification of mitochondrial elongation index and mitochondrial network aggregation in PV neurons (^*,#,$^*p* < 0.05 vs. vehicle, BSO and BSO + Ros-treated animals, *n* = 35 cells in 7 rats, respectively).

To induce oxidative stress under physiological condition, we applied BSO that inhibits γ-glutamylcysteine synthetase (a rate-limiting enzyme in GSH biosynthesis [27]) in normal rats. Western blot data showed that BSO decreased PV expression in the dentate gyrus, as compared to vehicle (*χ*^2^_(3)_ = 18.28, *p* < 0.05 vs. vehicle, *n* = 7, respectively, Kruskal-Wallis test with Dunn-Bonferroni post-hoc test; Fig. 1a, b and Supplementary Fig. 1). In addition, BSO diminished mitochondrial length in PV neurons without inducing PV neuronal degeneration (*χ*^2^_(3)_ = 78.45, *p* < 0.05 vs. vehicle, *n* = 7, respectively, Kruskal-Wallis test with Dunn-Bonferroni post-hoc test; Fig. 1c–e).

Because CDK5-mediated DRP1 S616 phosphorylation induces mitochondrial fragmentation in PV neurons [21, 28], we evaluated the effects of BSO on CDK5 Y15 and DRP1 S616 phosphorylations in the dentate gyrus. As compared to vehicle, BSO augmented CDK5 Y15 (*χ*^2^_(3)_ = 20.8, *p* < 0.05 vs. vehicle, *n* = 7, respectively, Kruskal-Wallis test with Dunn-Bonferroni post-hoc test) and DRP1 S616 phosphorylations (*χ*^2^_(3)_ = 15.37, *p* < 0.05 vs. vehicle, *n* = 7, respectively, Kruskal-Wallis test with Dunn-Bonferroni post-hoc test; Fig. 1c–e). To confirm the role of CDK5-DRP1 pathway in PV downregulation induced by BSO, we co-treated roscovitine (a CDK5 inhibitor) or Mdivi-1 (an inhibitor of mitochondrial fission) with BSO in normal rats. Both roscovitine and Mdivi-1 co-treatment attenuated BSO-induced PV downregulation and mitochondrial fission (Fig. 1a, b, d, e). However, Mdivi-1 did not affect CDK5 Y15 phosphorylation (Fig. 1a, b), but increased mitochondrial length as compared to roscovitine (*p* < 0.05 vs. roscovitine, *n* = 35 cells in 7 rats, respectively, Kruskal-Wallis test with Dunn-Bonferroni post-hoc test; Fig. 1d, e). These findings indicate that BSO-induced CDK5-DRP1-mediated mitochondrial fission may reduce PV expression under physiological condition without further CDK5 activation.

GPx1 scavenges ROS through the clearance of H_2_O_2_ by using GSH [29]. Since GPx1 downregulation is relevant to mitochondrial fission [30, 31], we also investigate whether BSO influences GPx1 expression in PV neurons. BSO diminished GPx1 expression in the dentate gyrus, which was attenuated by roscovitine or Mdivi-1 co-treatment (*χ*^2^_(3)_ = 15.44, *p* < 0.05 vs. vehicle, *n* = 7, respectively, Kruskal-Wallis test with Dunn-Bonferroni post-hoc test; Fig. 1a, b and Supplementary Fig. 1).

**Fig. 2:**
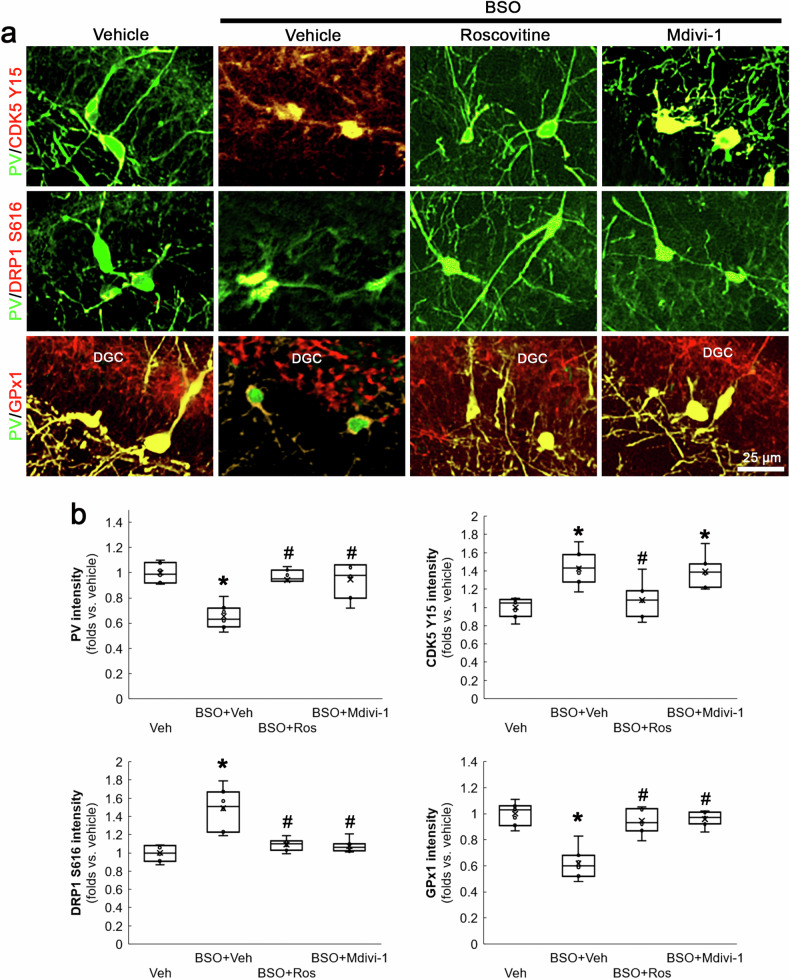
Effects of roscovitine and Mdivi-1 on BSO-induced PV downregulation, CDK5 and DRP1 hyperactivations and reduced GPx1 expression in PV neurons under physiological condition. BSO increases CDK5 Y15 and DRP1 S616 phosphorylations, concomitant with PV downregulation. However, BSO decreases GPx1 expression in PV neurons more than dentate granule cells (DGC), although GPx1 expression in PV neurons is higher than that in DGC in vehicle-treated rats. Roscovitine (Ros) attenuates CDK5 Y15 and DRP1 S616 hyperphosphorylations, PV downregulation and decreased GPx1 expression. Mdivi-1 ameliorates BSO-induced PV downregulation, DRP1 S616 phosphorylations and reduced GPx1 expression without altering CDK5 Y15 phosphorylation. **a** Representative images of CDK5 Y15, DRP1 S616 and GPx1 fluorescent intensity in PV neurons. **b** Quantification of CDK5 Y15, DRP1 S616 and GPx1 fluorescent intensity in PV neurons (^*,#^*p* < 0.05 vs. vehicle, BSO and BSO + Ros-treated animals, *n* = 7, respectively).

Immunohistochemical data revealed that increases in CDK5 Y15 and DRP1 S616 phosphorylation levels induced by BSO were mainly observed in PV neurons within the dentate gyrus (Fig. 2a, b). Under physiological condition, GPx1 expression in PV neurons was higher than that observed in dentate granule cells (DGC). BSO decreased GPx1 expression in PV neurons, concomitant with PV downregulation (Fig. 2a, b). BSO-induced changes in CDK5 Y15 (*χ*^2^_(3)_ = 17.33, *p* < 0.05 vs. vehicle, *n* = 7, respectively, Kruskal-Wallis test with Dunn-Bonferroni post-hoc test), DRP1 S616 (*χ*^2^_(3)_ = 17.25, *p* < 0.05 vs. vehicle, *n* = 7, respectively, Kruskal-Wallis test with Dunn-Bonferroni post-hoc test) and GPx1 (*χ*^2^_(3)_ = 15.78, *p* < 0.05 vs. vehicle, *n* = 7, respectively, Kruskal-Wallis test with Dunn-Bonferroni post-hoc test) levels in PV neurons were attenuated by roscovitine (Fig. 2a, b). Mdivi-1 ameliorated DRP1 S616 hyperphosphorylation and GPx1 downregulation without affecting augmented CDK Y15 phosphorylation (*p* < 0.05 vs. vehicle, *n* = 7, respectively, Kruskal-Wallis test with Dunn-Bonferroni post-hoc test; Fig. 2a, b). These findings indicate that CDK5-DRP1-mediated mitochondrial fission may augment PV and GPx1 downregulations induced by BSO-induced oxidative stress in PV neurons.

### Mitochondrial fission, not fusion, results in PV and GPx1 downregulations under physiological condition

**Fig. 3:**
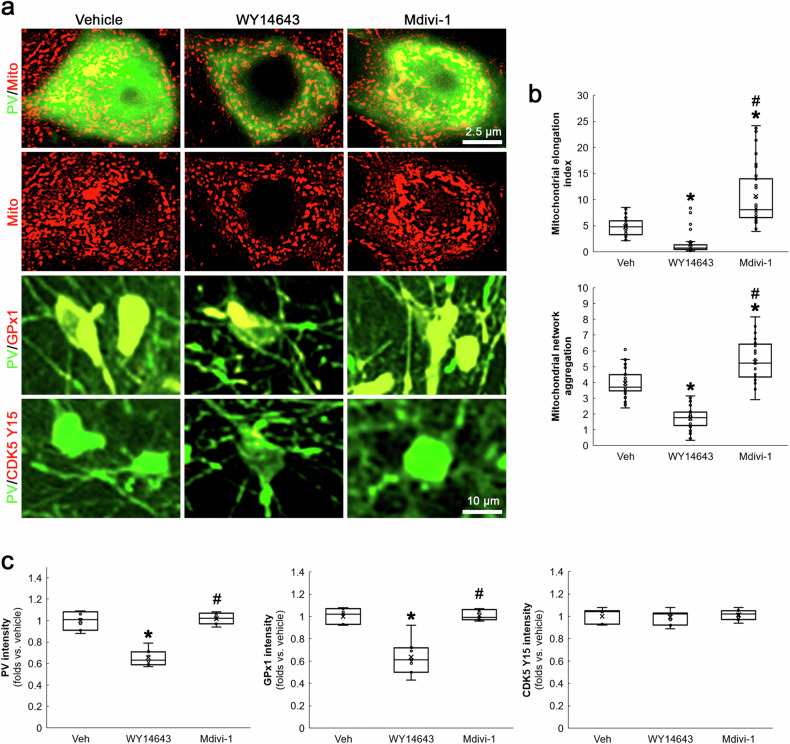
Effects of mitochondrial dynamics on PV, GPx1 and CDK5 Y15 fluorescent intensities in PV neurons under physiological condition. WY14643-induced mitochondrial fission decreases PV and GPx1 expressions without altering CDK5 Y15 phosphorylation in PV neurons. Mdivi-1-induced mitochondrial fusion cannot affect PV, GPx1 and CDK5 Y15 intensities in PV neurons. **a** Representative images of mitochondria, GPx1 expression and CDK5 Y15 phosphorylation in PV neurons. **b** Quantification of mitochondrial elongation index and mitochondrial network aggregation in PV neurons (^*,#^*p* < 0.05 vs. vehicle and WY14643-treated animals, *n* = 35 cells in 7 rats, respectively). **c** Quantification of GPx1 and CDK5 Y15 fluorescent intensity in PV neurons (^*,#^*p* < 0.05 vs. vehicle and WY14643-treated animals, *n* = 7, respectively).

To further confirm the role of mitochondrial fission in PV downregulation, we applied WY14643 (an inducer of mitochondrial fission) or Mdivi-1 in normal rats. Compatible with previous studies [22, 32], WY14643 led to mitochondrial fission (*χ*^2^_(2)_ = 69.578, *p* < 0.05 vs. vehicle, *n* = 35 cells in 7 rats, respectively, Kruskal-Wallis test with Dunn-Bonferroni post-hoc test) and PV downregulation (*χ*^2^_(2)_ = 13.4, *p* < 0.05 vs. vehicle, *n* = 7, respectively, Kruskal-Wallis test with Dunn-Bonferroni post-hoc test) in PV neurons (Fig. 3a–c). Mdivi-1 did not alter PV expression, while it elongated mitochondrial length (*p* < 0.05 vs. vehicle, *n* = 35 cells in 7 rats, respectively, Kruskal-Wallis test with Dunn-Bonferroni post-hoc test; Fig. 3a–c). In addition, WY14643, not Mdivi-1, decreased GPx1 expression level in PV neurons (*χ*^2^_(2)_ = 13.18, *p* < 0.05 vs. vehicle, *n* = 7, respectively, Kruskal-Wallis test with Dunn-Bonferroni post-hoc test; Fig. 3a, c). Neither WY14643 nor Mdivi-1 altered CDK5 Y15 phosphorylation in PV neurons (Fig. 3a, c). Together with the data obtained from BSO treatment, our findings indicate that mitochondrial fission itself may diminish PV and GPx1 expressions under physiological condition, which would not affect CDK5 Y15 phosphorylation in a positive feedback manner.

### NAC attenuates SE-induced PV neuronal degeneration by inhibiting CDK5 Y15 hyperphosphorylation, excessive mitochondrial fragmentation and reduced GPx1-mediated NF-κB inhibition

**Fig. 4:**
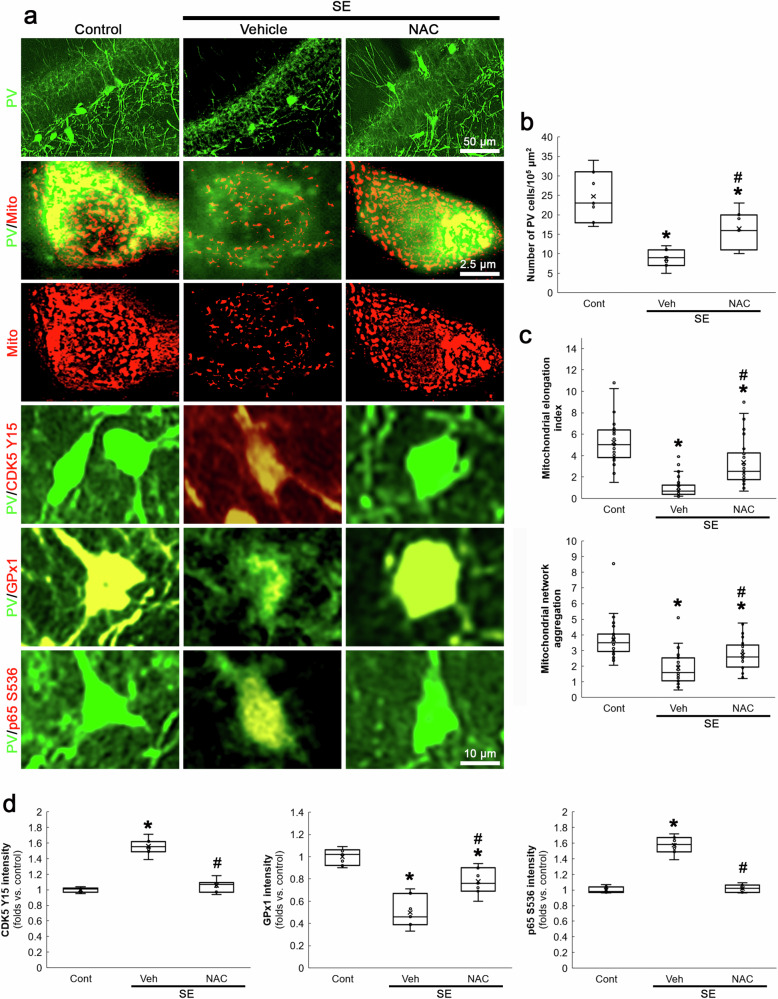
Effects of NAC on PV cell degeneration, mitochondrial fragmentation, CDK5 Y15, GPx1 and p65 S536 fluorescent intensities in PV neurons following SE. SE decreases the number of PV neurons in the dentate gyrus, accompanied by mitochondrial fragmentation. SE increases CDK5 Y15 and p65 S536 phosphorylation, while it reduces GPx1 expression. NAC ameliorates these post-SE events. **a** Representative images of PV neurons, mitochondria, CDK5 Y15 phosphorylation, GPx1 expression and p65 S536 phosphorylation in PV neurons. **b** Quantification of the number of PV neurons in the dentate gyrus (^*,#^*p* < 0.05 vs. control and vehicle-treated animals, *n* = 7, respectively). **c** Quantification of mitochondrial elongation index and mitochondrial network aggregation in PV neurons (^*,#^*p* < 0.05 vs. control and vehicle-treated animals, *n* = 35 cells in 7 rats, respectively). **d** Quantification of CDK5 Y15, GPx1 and p65 S536 fluorescent intensity in PV neurons (^*,#^*p* < 0.05 vs. control and vehicle-treated animals, *n* = 7, respectively).

SE-induced NADPH oxidase activation exerts PV downregulation and the subsequent PV neuronal loss, accompanied by CDK5-DRP1-mediated mitochondrial fragmentation [21]. NADPH oxidase contributes to ROS generation induced by SE [33, 34] and its upregulation is predominantly observed in PV neurons following SE [21]. Therefore, it is likely that oxidative stress would lead CDK5 hyperactivation and aberrant mitochondrial fragmentation in PV neurons following SE. Compatible with our previous study [21], the present data showed that SE led to PV neuronal degeneration in the hilus of the dentate gyrus (*χ*^*2*^_(2)_ = 14.17, *p* < 0.05 vs. control, *n* = 7, respectively, Kruskal-Wallis test with Dunn-Bonferroni post-hoc test; Fig. 4a, b), accompanied by mitochondrial fragmentation (*χ*^2^_(2)_ = 62.1, *p* < 0.05 vs. control, *n* = 35 cells in 7 rats, respectively, Kruskal-Wallis test with Dunn-Bonferroni post-hoc test; Fig. 4a, c) and CDK5 Y15 hyperphosphorylation (*χ*^2^_(2)_ = 14.38, *p* < 0.05 vs. control, *n* = 7, respectively, Kruskal-Wallis test with Dunn-Bonferroni post-hoc test; Fig. 4a, d). To validate the role of oxidative stress in SE-induced PV neuronal degeneration, we evaluated the effects of NAC (an antioxidant) on post-SE events. As compared to vehicle, NAC attenuated PV cell degeneration (*p* < 0.05 vs. vehicle, *n* = 7, respectively; Fig. 4a, b), mitochondrial fragmentation (*p* < 0.05 vs. vehicle, *n* = 35 cells in 7 rats, respectively; Fig. 4a, c) and CDK5 Y15 hyperphosphorylation (*p* < 0.05 vs. vehicle, *n* = 7, respectively; Fig. 4a, d) following SE.

On the other hand, reduced GPx1 expression enhances NF-κB p65 S536 phosphorylation, which is closely relevant to cell degeneration [35–38]. Thus, we also validated the effect of NAC on SE-induced p65 S536 phosphorylation in PV neurons. Consistent with a previous study [35], NAC mitigated GPx1 downregulation (*χ*^2^_(2)_ = 15.89, *p* < 0.05 vs. vehicle, *n* = 7, respectively, Kruskal-Wallis test with Dunn-Bonferroni post-hoc test; Fig. 4a, d) and abolished enhanced p65 S536 phosphorylation (*χ*^2^_(2)_ = 13.64, *p* < 0.05 vs. vehicle, *n* = 7, respectively, Kruskal-Wallis test with Dunn-Bonferroni post-hoc test; Fig. 4a, d) in PV neurons following SE. These findings suggest that oxidative stress may play a crucial role in SE-induced PV neuronal degeneration through CDK5-DRP1- and GPx1-NF-κB-mediated signaling pathways in PV neurons.

### CDK5 inhibition rescues PV neurons and inhibits GPx1-NF-κB signaling pathway following SE

**Fig. 5:**
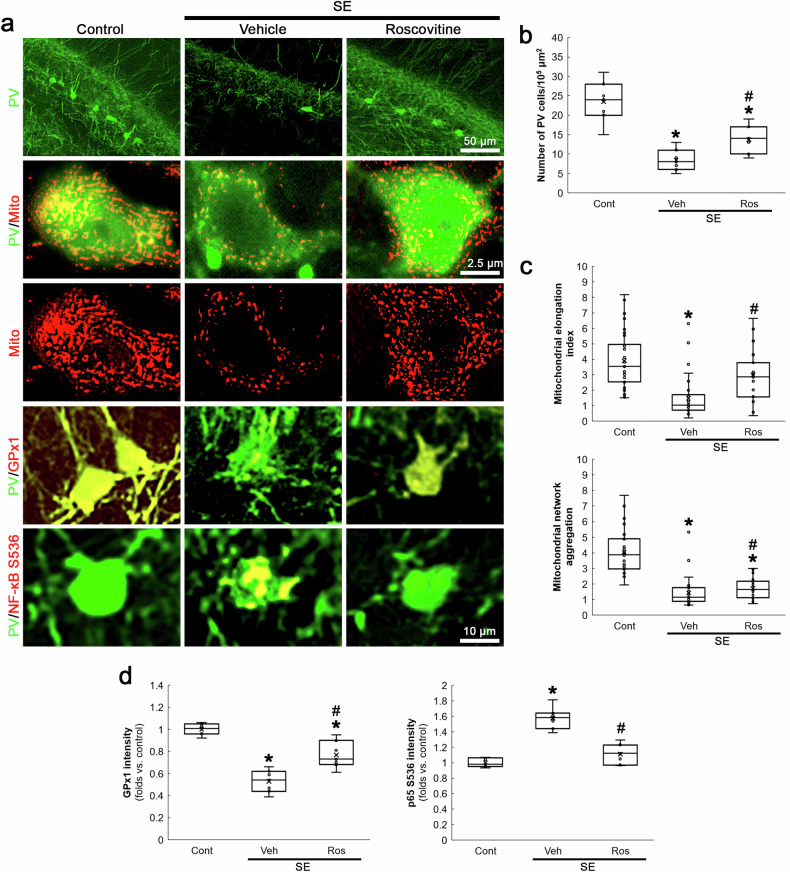
Effects of roscovitine on PV cell degeneration, mitochondrial fragmentation, GPx1 and p65 S536 fluorescent intensities in PV neurons following SE. Roscovitine (Ros) attenuates SE-induced PV neuronal degeneration, mitochondrial fragmentation, GPx1 downregulation and p65 S536 phosphorylation in PV neurons. **a** Representative images of PV neurons, mitochondria, GPx1 expression and CDK5 Y15 phosphorylation in PV neurons. **b** Quantification of the number of PV neurons in the dentate gyrus (^*,#^*p* < 0.05 vs. control and vehicle-treated animals, *n* = 7, respectively). **c** Quantification of mitochondrial elongation index and mitochondrial network aggregation in PV neurons (^*,#^*p* < 0.05 vs. control and vehicle-treated animals, *n* = 35 cells in 7 rats, respectively). **d** Quantification of GPx1 and p65 S536 fluorescent intensity in PV neurons (^*,#^*p* < 0.05 vs. control and vehicle-treated animals, *n* = 7, respectively).

Although GPx1 downregulation is relevant to mitochondrial fission [30, 31], the interaction or relationship between CDK5 and GPx1 has been elusive. However, the present study revealed that NAC attenuated SE-induced PV neuronal loss induced by aberrant mitochondrial fission, concomitant with GPx1 upregulation as well as reduced p65 S536 phosphorylation (Fig. 4). Therefore, we further explored whether CDK5 hyperactivation affects GPx1-NF-κB signaling pathway following SE. In the present study, roscovitine attenuated PV neuronal degeneration (*χ*^2^_(2)_ = 15.44, *p* < 0.05 vs. vehicle, *n* = 7, respectively, Kruskal-Wallis test with Dunn-Bonferroni post-hoc test; Fig. 5a, b) and mitochondrial fragmentation in PV neurons following SE (*χ*^2^_(2)_ = 37.76, *p* < 0.05 vs. vehicle, *n* = 35 cells in 7 rats, respectively, Kruskal-Wallis test with Dunn-Bonferroni post-hoc test; Fig. 5a, c). Roscovitine also attenuated GPx1 downregulation (*χ*^2^_(2)_ = 16.75, *p* < 0.05 vs. vehicle, *n* = 7, respectively, Kruskal-Wallis test with Dunn-Bonferroni post-hoc test; Fig. 5a, d) and p65 S536 phosphorylation (*χ*^2^_(2)_ = 14.63, *p* < 0.05 vs. vehicle, *n* = 7, respectively, Kruskal-Wallis test with Dunn-Bonferroni post-hoc test; Fig. 5a, d) induced by SE. These findings indicate that CDK5 hyperactivation may be involved in GPx1-mediated NF-κB inhibition following SE.

### Inhibition of mitochondrial fission ameliorates CDK5 Y15 hyperphosphorylation, GPx1 downregulation and p65 S536 phosphorylation in PV neurons following SE

**Fig. 6:**
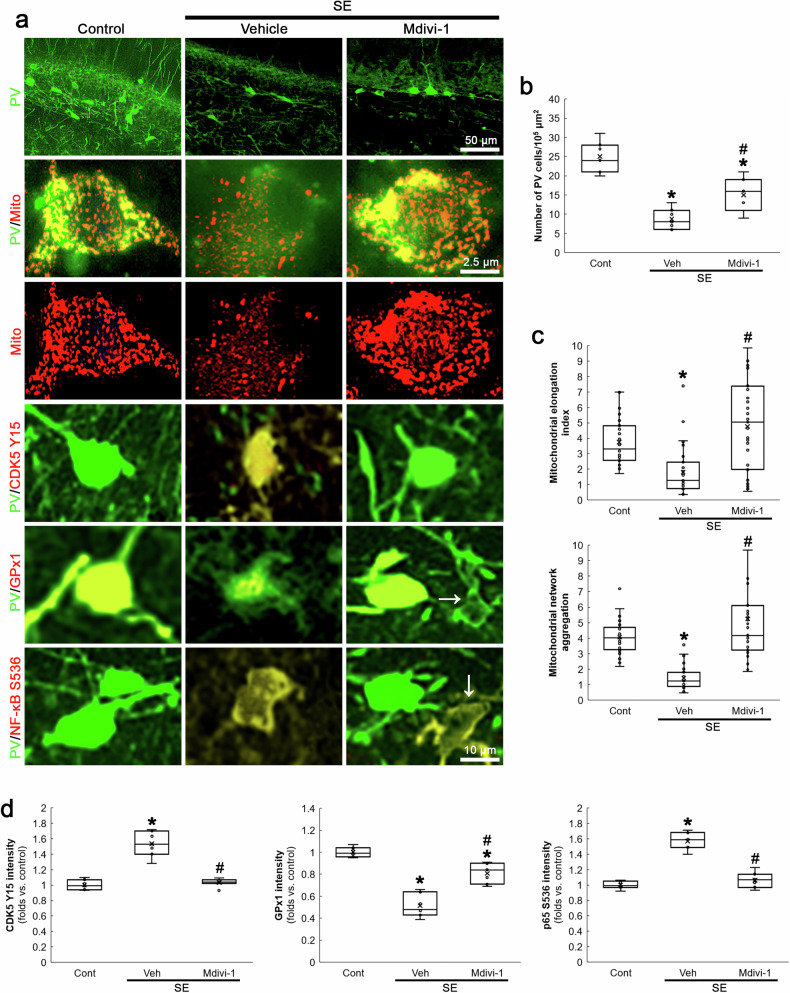
Effects of Mdivi-1 on PV cell degeneration, mitochondrial fragmentation, CDK5 Y15, GPx1 and p65 S536 fluorescent intensities in PV neurons following SE. Mdivi-1 ameliorates SE-induced PV neuronal degeneration, mitochondrial fragmentation, CDK5 Y15 phosphorylation, GPx1 downregulation and p65 S536 phosphorylation in PV neurons. **a** Representative images of PV neurons, mitochondria, CDK5 Y15 phosphorylation, GPx1 expression and p65 S536 phosphorylation in PV neurons. **b** Quantification of the number of PV neurons in the dentate gyrus (^*,#^*p* < 0.05 vs. control and vehicle-treated animals, *n* = 7, respectively). **c** Quantification of mitochondrial elongation index and mitochondrial network aggregation in PV neurons (^*,#^*p* < 0.05 vs. control and vehicle-treated animals, *n* = 35 cells in 7 rats, respectively). **d** Quantification of CDK5 Y15, GPx1 and p65 S536 fluorescent intensity in PV neurons (^*,#^*p* < 0.05 vs. control and vehicle-treated animals, *n* = 7, respectively).

Compatible with previous studies [28, 39], the present data demonstrated that CDK5 inhibition abolished DRP1-mediated mitochondrial fission in PV neurons. Although the present data showed that mitochondrial fission could not affect BSO-induced CDK5 activation in PV neurons under physiological condition (Figs. 1 and 2), the role of mitochondrial fragmentation in CDK5 activity under post-SE condition has been unreported. Thus, we investigated if aberrant mitochondrial fragmentation would affect CDK5 activation and GPx1-mediated NF-κB inhibition following SE. In the present study, Mdivi-1 reduced SE-induced PV neuronal degeneration (*χ*^2^_(2)_ = 15.66, *p* < 0.05 vs. vehicle, *n* = 7, respectively, Kruskal-Wallis test with Dunn-Bonferroni post-hoc test; Fig. 6a, b). In addition, Mdivi-1 ameliorated excessive mitochondrial fragmentation (*χ*^2^_(2)_ = 28.97, *p* < 0.05 vs. vehicle, *n* = 35 cells in 7 rats, respectively, Kruskal-Wallis test with Dunn-Bonferroni post-hoc test; Fig. 6a, c), CDK5 Y15 hyperphosphorylation (*χ*^2^_(2)_ = 13.75, *p* < 0.05 vs. vehicle, *n* = 7, respectively, Kruskal-Wallis test with Dunn-Bonferroni post-hoc test; Fig. 6a, d), GPx1 downregulation (*χ*^2^_(2)_ = 17.83, *p* < 0.05 vs. vehicle, *n* = 7, respectively, Kruskal-Wallis test with Dunn-Bonferroni post-hoc test; Fig. 6a, d) and increased p65 S536 phosphorylation (*χ*^2^_(2)_ = 14.2, *p* < 0.05 vs. vehicle, *n* = 7, respectively, Kruskal-Wallis test with Dunn-Bonferroni post-hoc test; Fig. 6a, d) in PV neurons following SE. Since Mdivi-1 inhibits mitochondrial fission [39, 40] and reduces mitochondrial ROS generation [41, 42], our findings indicate that SE-induced mitochondrial fission may augment mitochondrial ROS generation and subsequently exacerbate oxidative stress, which would further increase CDK5 Y15 phosphorylation in a positive feedback manner and subsequently lead to GPx1 downregulation and p65 S536 phosphorylation during PV neuronal degeneration.

### NF-κB inhibition mitigates SE-induced PV neuronal degeneration independent of aberrant mitochondrial fission

**Fig. 7:**
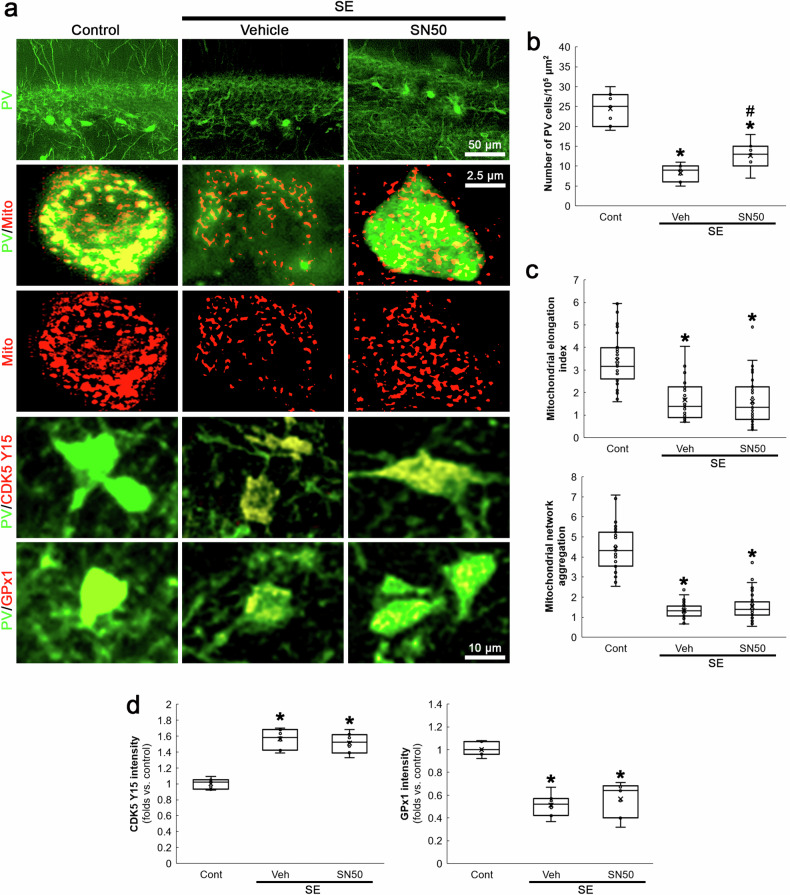
Effects of SN50 on PV cell degeneration, mitochondrial fragmentation, CDK5 Y15 and GPx1 fluorescent intensities in PV neurons following SE. SN50 mitigates SE-induced PV neuronal degeneration without affecting mitochondrial fragmentation, CDK5 Y15 phosphorylation and GPx1 downregulation in PV neurons. **a** Representative images of PV neurons, mitochondria, CDK5 Y15 phosphorylation and GPx1 expression in PV neurons. **b** Quantification of the number of PV neurons in the dentate gyrus (^*,#^*p* < 0.05 vs. control and vehicle-treated animals, *n* = 7, respectively). **c** Quantification of mitochondrial elongation index and mitochondrial network aggregation in PV neurons (^*,#^*p* < 0.05 vs. control and vehicle-treated animals, *n* = 35 cells in 7 rats, respectively). **d** Quantification of CDK5 Y15 and GPx1 fluorescent intensity in PV neurons (^*,#^*p* < 0.05 vs. control and vehicle-treated animals, *n* = 7, respectively).

Oxidative stress activates NF-κB signaling pathway that induces PV downregulation [43]. Furthermore, Mdivi-1 inhibits NF-κB activity [40] and mitochondrial fission reciprocally modulates NF-κB pathway, which are linked to increased oxidative stress [44, 45]. The present study also revealed that Mdivi-1 abrogated GPx1 downregulation and p65 S536 phosphorylation in PV neurons following SE (Fig. 6). Thus, we explored whether NF-κB activation influences SE-induced mitochondrial fission in PV neurons. As compared to vehicle, SN50 (a NF-κB inhibitor) attenuated SE-induced PV neuronal degeneration (*χ*^2^_(2)_ = 15.67, *p* < 0.05 vs. vehicle, *n* = 7, respectively, Kruskal-Wallis test with Dunn-Bonferroni post-hoc test; Fig. 7a, b). However, it could not affect mitochondrial fragmentation, CDK5 Y15 phosphorylation and GPx1 downregulation following SE (Fig. 7a, c, d). These findings suggest that NF-κB may be a downstream effector of CDK5-DRP1-mediated mitochondrial fragmentation event, which would elicit oxidative stress-induced PV neuronal degeneration following SE.

## Discussion

**Fig. 8:**
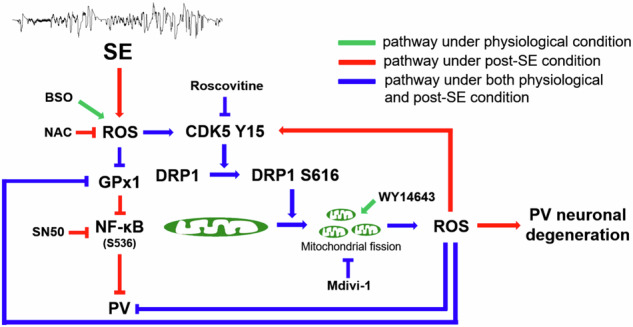
Schematic depiction representing the reciprocal regulation of oxidative stress and mitochondrial fission on PV expression through CDK5-DRP1- and GPx1-NF-κB signaling pathways. Under physiological condition, BSO-induced oxidative stress facilitates CDK5-DRP1-mediated mitochondrial fission in PV neurons, which subsequently diminishes PV and GPx1 expressions. Mitochondrial fission-induced oxidative stress also affectes PV and GPx1 expressions. Following SE, mitochondrial fission contributed to PV downregulation and PV neuronal degeneration through CDK5-DRP1- and GPx1-NF-κB-mediated signaling pathways.

The novel findings in the present study are ROS-CDK5-DRP1-mediated mitochondrial fission reduced PV expression under physiological condition and also led to PV neuronal loss by abolishing GPx1-mediated NF-κB inhibition following SE. These findings indicate that mitochondrial fission may regulate PV expression and GPx1-mediated NF-κB signaling pathway, which would play an important role in SE-induced PV neuronal degeneration (Fig. 8).

PV is an intracellular modulator capable of Ca^2+^ sequestering/buffering. Similar to PV, mitochondria also uptake large amounts of Ca^2+^ ions as a regulator/decoder of Ca^2+^ signals [46]. Thus, PV prevents mitochondrial Ca^2+^ overload and mitochondria replace Ca^2+^ buffering capacity provided by PV when PV expression is reduced [47, 48]. Indeed, PV ablation directly leads to increased mitochondrial volume in PV neurons [18–20], while PV overexpression reduces mitochondrial volume and mitochondrial fusion rates [19, 49, 50]. Therefore, it is likely that the antagonistic regulation between PV expression and mitochondrial dynamics would operate in a bidirectional and homeostatic manner. Although adequate PV presence is essential for maintenance of mitochondrial functions, the effect of mitochondrial dynamics on PV expression has not been directly addressed in vivo. In the present study, mitochondrial fission induced by WY14643 led to PV downregulation, while fusion mediated by Mdivi-1 did not affect PV expression level under physiological condition. These findings indicate that mitochondrial fragmentation itself may also result in PV downregulation. How does mitochondrial fragmentation underlie PV downregulation under physiological condition? Fragmentated mitochondria show an insufficient capacity of Ca^2+^ buffering [51] and a decline in mitochondrial membrane potential, which elevate mitochondrial ROS generation [52, 53]. Indeed, GSH deprivation reduces the number of PV neurons, indicating the deleterious effect of oxidative stress on PV expression [24, 25]. Furthermore, mitochondrial fission itself increases reverse electron transfer-mediated ROS burden [41, 54, 55]. The present study also demonstrates that BSO resulted in PV downregulation, which was abrogated by inhibiting mitochondrial fission with roscovitine or Mdivi-1 co-treatment. Therefore, our findings suggest that redox dysregulation may lead to mitochondrial fission, which would further increase ROS level diminishing PV expression under physiological condition.

The fast-firing PV neurons play fundamental roles in the regulation of neuronal synchrony (particularly in γ-oscillation) and GABAergic inhibitory neurotransmission [5, 25]. The maintenance of PV neuronal functions, such as fast-spiking properties and rapid action potential kinetics, requires the high bioenergetics by optimal mitochondrial functions [56, 57]. Thus, the precise regulation of mitochondrial dynamics in PV neurons is important for proper neuronal signaling and network synchronization [58]. Furthermore, PV neurons show the higher mitochondrial ROS generation during oxidative phosphorylation under physiological condition that renders them highly vulnerable to oxidative stress associated with deficiency of energy substrates or mitochondrial dysfunction [5, 57]. The present study also demonstrates that GPx1 expression level in PV neurons was higher than that in DGC under physiological condition. Therefore, PV neurons are involved in the pathogenesis of various brain diseases relevant to neuronal network dysfunction and/or metabolism dysregulation. In pathological conditions, the activation of ionotropic glutamate receptors with high Ca^2+^-permeability and/or reduced PV expression results in cytosolic and mitochondrial Ca^2+^-overloads that enhance ROS generation. Thus, the acquired PV downregulation and/or PV neuronal degeneration are thought to underlie pathophysiological mechanisms of various neurological and psychiatric disorders [6]. Indeed, SE provokes PV neuronal degeneration accompanied by CDK5-mediated mitochondrial fragmentation, which is induced by NADPH oxidase-induced oxidative stress [21]. Compatible with these reports, the present study reveals that unlike BSO treatment SE led to PV downregulation and excessive mitochondrial fission in PV neurons through CDK5-mediated DRP1 S616 phosphorylation in a positive feedback manner, which were attenuated by NAC, roscovitine or Mdivi-1. When PV neurons are activated, Ca^2+^ influx is faster, larger and longer than that in excitatory principal neurons [59]. This bulk Ca^2+^ influx into neurons triggers intracellular Ca^2+^ overload and CDK5 activation, which lead to excessive DRP1-mediated mitochondrial fission [60]. Subsequently, this Ca^2+^-induced mitochondrial fragmentation increases ROS formation and evokes PV downregulation [25, 61], which further deteriorates oxidative stress and accelerates mitochondrial fragmentation independent of mitochondrial Ca^2+^ uptake [18–20, 62, 63]. Therefore, our findings suggest that mitochondrial fission may not only be a consequence from PV neuronal hyperactivation in response to SE but also be involved in ROS generation and PV downregulation as post-SE events, which would further exacerbate impaired Ca^2+^ buffering and mitochondrial dysfunction/fragmentation, thereby finally leading to PV neuronal degeneration.

On the other hand, the present study reveals that BSO decreased GPx1 expression in PV neurons under physiological condition, which was attenuated by roscovitine or Mdivi-1 co-treatment. GPx1 plays an important role in clearance of H_2_O_2_ [29], and GPx1 downregulation induces mitochondrial fission [30, 31]. Thus, the present data indicate that mitochondrial fission-induced ROS generation may reinforce PV and GPx1 downregulations induced by BSO. Similar to BSO treatment, the present study demonstrates that SE significantly reduced GPx1 expression in PV neurons concomitant with excessive mitochondrial fission. In addition, SE-induced GPx1 downregulation was alleviated by roscovitine, Mdivi-1 or NAC. Therefore, it is likely that SE-induced GPx1 downregulation may directly enable increased ROS generation and mitochondrial fission and further aggravate oxidative stress in PV neurons. In addition, oxidative stress-induced GPx1 downregulation increases NF-κB activity that decreases cell viability, PV expression and mitochondrial length [35–38, 43–45]. The present study demonstrates that NAC, roscovitine or Mdivi-1 treatment ameliorated SE-induced GPx1 downregulation and p65 S536 phosphorylation in PV neurons. In contrast, SN50 attenuated SE-induced PV neuronal degeneration, independent of CDK5 Y15 hyperphosphorylation, mitochondrial fragmentation and GPx1 downregulation. Therefore, our findings suggest that GPx1-NF-κB-mediated signaling pathway may be one of downstream of CDK5-DRP1-mediated mitochondrial fragmentation events, which would lead to oxidative stress-induced PV neuronal degeneration following SE.

## Conclusion

In the present study, we assessed the role of mitochondrial fission in PV expression under physiological and post-SE conditions. As the results, we found that BSO-induced oxidative stress led to CDK5-DRP1-mediated mitochondrial fission in PV neurons, which subsequently diminished PV expression under physiological condition. In addition, induction of mitochondrial fission resulted in PV and GPx1 downregulations without altering CDK5 Y15 phosphorylation. Following SE, mitochondrial fission contributed to CDK5 hyperactivation, PV downregulation and PV neuronal degeneration through GPx1 downregulation and NF-κB activation. These findings overall suggest that oxidative stress and aberrant mitochondrial fission may reciprocally regulate each other in PV neurons through CDK5-DRP1- and GPx1-NF-κB-mediated signaling pathways, which would be important to maintain the phenotype, functionality and viability of PV neurons (Fig. 8). Therefore, future studies are needed to develop the therapeutic techniques for manipulation of PV expression in neurological and psychiatric diseases.

## Materials and methods

### Experimental animals and chemicals

One hundred twenty-five adult male Sprague-Dawley (SD) rats (7 weeks old) were used in the present study. Animals were randomly allocated to (1) oxidative stress induction group (BSO treatment; *n* = 56), (2) mitochondrial dynamics induction group (WY14643- or Mdivi-1 treatment without BSO treatment and SE induction; *n* = 14) and (3) SE induction group (*n* = 49). Six rats died during SE induction. Each experimental group was also divided into subgroups as described in Surgery, BSO treatment and SE induction sections (see below). Animals were kept under controlled environmental conditions (23–25 °C, 12 h light/dark cycle) to access to water and food *ad libitum* throughout the experiments. All experimental protocols described below were approved by the Institutional Animal Care and Use Committee of Hallym University (Hallym 2021-30, approval date: 17, May. 2021). All reagents were obtained from Sigma-Aldrich (St. Louis, MO, USA), except as noted.

### Surgery

Rats were implanted with an infusion needle (brain infusion kit 1; Alzet, Cupertino, CA, USA) into the right lateral ventricle (coordinates: 1 mm posterior; 1.5 mm lateral; 3.5 mm depth) under isoflurane anesthesia (3% induction, 1.5–2% for surgery and 1.5% maintenance in a 65:35 mixture of N_2_O:O_2_), followed by connecting an osmotic pump (1007D; Alzet, Cupertino, CA, USA) containing (1) vehicle (*n* = 14 for BSO treatment and *n* = 7 for SE induction, respectively), (2) roscovitine (100 μM, *n* = 14 for BSO treatment and *n* = 7 for SE induction, respectively), (3) WY14643 (150 μM, *n* = 7 without BSO treatment and SE induction), (4) Mdivi-1 (50 μM, *n* = 14 for BSO treatment, *n* = 7 for SE induction and *n* = 7 without BSO treatment and SE induction, respectively) or (5) SN50 (20 μM, *n* = 7 for SE induction). Each treatment did not evoke neurological adverse effects or alter the seizure susceptibility and its severity in response to pilocarpine [21, 22, 32, 64].

### BSO treatment

Two days after surgery, BSO (1.3 g/kg, i.p.) was administered once a day during 3 days (*n* = 42) [65, 66]. Some animals were given vehicle by the same methods (*n* = 14). Five hours after the last injection, animals were used for Western blot and immunohistochemistry.

### SE induction

Two days after surgery, some rats were subjected to the LiCl-pilocarpine model of temporal lobe epilepsy (TLE). Rats were given LiCl (127 mg/kg, i.p.) 24 h before the pilocarpine treatment. Animals were treated with pilocarpine (30 mg/kg, i.p.) 20 min after atropine methylbromide (5 mg/kg i.p.). Two hours after status epilepticus (SE) onset, diazepam (Valium; Hoffmann-la Roche, Neuilly-sur-Seine, France; 10 mg/kg, i.p.) was administered to cease SE and repeated, as needed. Control animals received saline in place of pilocarpine (*n* = 7). Some animals were administered vehicle (*n* = 7) or NAC (70 mg/kg, i.p., *n* = 7) 2 days before SE induction once a day during 5 days [65, 66]. Three days after SE, animals were used for immunohistochemistry.

### Western blot

Rats were decapitated under urethane anesthesia (1.5 g/kg, i.p.; *n* = 7 in each group respectively, total number = 28). The brain was quickly removed and coronally sliced 1 mm thickness (approximately 3–4 mm posterior to the bregma) using a rodent brain matrix (World Precision Instruments, Sarasota, FL, USA) on ice. Thereafter, the brain slice was placed in cold (4 °C) artificial cerebrospinal fluid. A sharp needle tip was inserted into the border between the dentate gyrus and the hippocampal proper (the hippocampal fissure) within the dorsal hippocampus under a stereomicroscope. The CA1 region was first separated from the dentate gyrus through the hippocampal fissure along the septo-temporal axis of the hippocampus, and then removed the CA3 region from the hilus of the dentate gyrus along the dorso-ventral axis of the dentate gyrus. The individual dentate gyrus obtained from each animal was homogenized in lysis buffer. The protein concentration in the supernatant was determined using a Micro BCA Protein Assay Kit (Pierce Chemical, Dallas, TX, USA). Thereafter, Western blot was performed in duplicate for each dentate gyrus by the standard protocol (*n* = 7 in each group). The primary antibodies used in the present study are listed in Table 1. The bands were detected and quantified on an ImageQuant LAS4000 system (GE Healthcare Korea, Seoul, South Korea). As an internal reference, rabbit anti-β-actin primary antibody (1:5000) was used. The values of each sample were normalized with the corresponding amount of β-actin [21, 22, 32, 66].

**Table 1:** Primary antibodies used in the present study.

Antigen	Host	Manufacturer (catalog number)	Dilution used
GPx1	Goat Sheep	R&D systems (#AF3798) Biosensis (#S-072-100)	1:1000 (WB) 1:2000 (IH)
PV	Goat	Swant (#PVG-213)	1:1000 (WB) 1:1000 (IH)
NF-κB p65	Rabbit	Abcam (#ab16502)	1:2000 (WB)
NF-κB p65 S536	Rabbit	Abcam (#ab28856)	1:1000 (WB) 1:100 (IH)
DRP1	Rabbit	Thermo (#PA1-16987)	1:1000 (WB)
DRP1 S616	Rabbit	Cell Signaling (#4494)	1:1000 (WB) 1:500 (IH)
CDK5	Rabbit	Abcam (#ab40773)	1:5,000 (WB)
Cleaved (active) caspase-3	Rabbit	Cell Signaling (#9664)	1:500 (IH)
CDK5 Y15	Rabbit	GeneTex (#GTX32375)	1:1000 (WB) 1:100 (IH)
Mitochondrial marker (mitochondrial complex IV subunit 1, MTCO1)	Mouse	Abcam (#ab14705)	1:500 (IH)
β-actin	Mouse	Sigma (#A5316)	1:5000 (WB)

*IH* immunohistochemistry, *WB* Western blot.

### Immunohistochemistry and mitochondrial morphometry

Rats were anesthetized with urethane (1.5 g/kg, i.p.; *n* = 7 in each group, respectively) and perfused transcardially with 4% paraformaldehyde in 0.1 M phosphate buffer (PB, pH 7.4). Brains were post-fixed in the same fixative overnight. Brain tissues were cryoprotected by infiltration with 30% sucrose overnight. Thereafter, Brains were cryosectioned at 30 μm. Free-floating sections were washed 3 times in PBS (0.1 M, pH 7.3) and incubated with 3% bovine serum albumin in PBS for 30 min at room temperature. Later, sections were incubated with a cocktail solution containing primary antibodies (Table 1) in PBS containing 0.3% Triton X-100 overnight at room temperature. Sections were visualized with appropriate Cy2- or Cy3-conjugated secondary antibodies. To establish the specificity of the immunostaining, a negative control test was carried out with preimmune serum instead of the primary antibody. All experimental procedures in this study were performed under the same conditions and in parallel. To measure fluorescent intensity, 5 sections of the dentate gyrus were chosen from each animal (*n* = 7 rats in each group) and then an area of interest (300 μm^2^/area) were randomly selected in each slice. Thereafter, mean intensity on 5 sections from each animal was measured by using AxioVision Rel. 4.8 and ImageJ software. Intensity measurements were represented as the number of a 256 gray scale. Intensity of each section was standardized by setting the threshold level (mean background intensity obtained from five image inputs). Manipulation of the images was restricted to threshold and brightness adjustments to the whole image. To count PV neurons, sections (5 sections per each animal) were captured and areas of interest (1 × 10^4^ μm^2^) were selected from the dentate gyrus using an AxioImage M2 microscope. Thereafter, PV cell counts were performed using AxioVision Rel. 4.8 Software [21, 22, 32, 66]. Mitochondrial morphometry was analyzed from thirty-five PV neurons randomly selected in the dentate gyrus (5 sections from each animal; *n* = 7 rats in each group). To conduct mitochondrial morphometry, the mitochondrial elongation index (area-weighted form factor = perimeter^2^/4*π*) and mitochondrial network aggregation (cumulative area:perimeter ratio = Σarea/Σperimeter) in these neurons were calculated using ImageJ software. The mitochondrial elongation index indicates the increased mitochondrial length (mitochondrial elongation) by the transition from a punctiform to an elongated shape. In addition, mitochondrial network aggregation (cumulative area:perimeter ratio) indicates the transition from elongated, isolated mitochondria to a reticular network or the aggregation of interconnected mitochondria [67, 68]. Investigators who performed the fluorescent intensity measurements, PV cell count and mitochondrial morphometry were blinded to the classification of tissues.

### Data analysis

To analyze statistical significance of data, Mann-Whitney test and Kruskal-Wallis test with Dunn-Bonferroni post-hoc test were applied. A *p*-value less than 0.05 was considered to be significant.

## Supplementary information

Supplementary information

Supplementary Figure 1. Full-gel images of Western blot in Figure 1

aj-checklist

## Data Availability

No data was used for the research described in the article. All data generated or analyzed during this study are included in this published article and its Supplementary Information files.
