# Correction: Reciprocal regulation of oxidative stress and mitochondrial fission augments parvalbumin downregulation through CDK5-DRP1- and GPx1-NF-κB signaling pathways

**DOI:** 10.1038/s41419-025-08409-y

**Published:** 2026-01-28

**Authors:** Su Hyeon Wang, Duk-Shin Lee, Tae-Hyun Kim, Ji-Eun Kim, Tae-Cheon Kang

**Affiliations:** Department of Anatomy and Neurobiology, Institute of Epilepsy Research, College of Medicine, Hallym University, Chuncheon, South Korea

**Keywords:** Epilepsy, Cell death in the nervous system

Correction to: *Cell Death & Disease* 10.1038/s41419-024-07050-5, published online 30 September 2024

The authors regret to inform that an error was present in Fig. 6a (SE + Mdivi-1 image) of the original article. All authors have reviewed and approved this correction. We apologize for the error and confirm that this correction does not alter the results or conclusion of the article.

Original Fig. 6
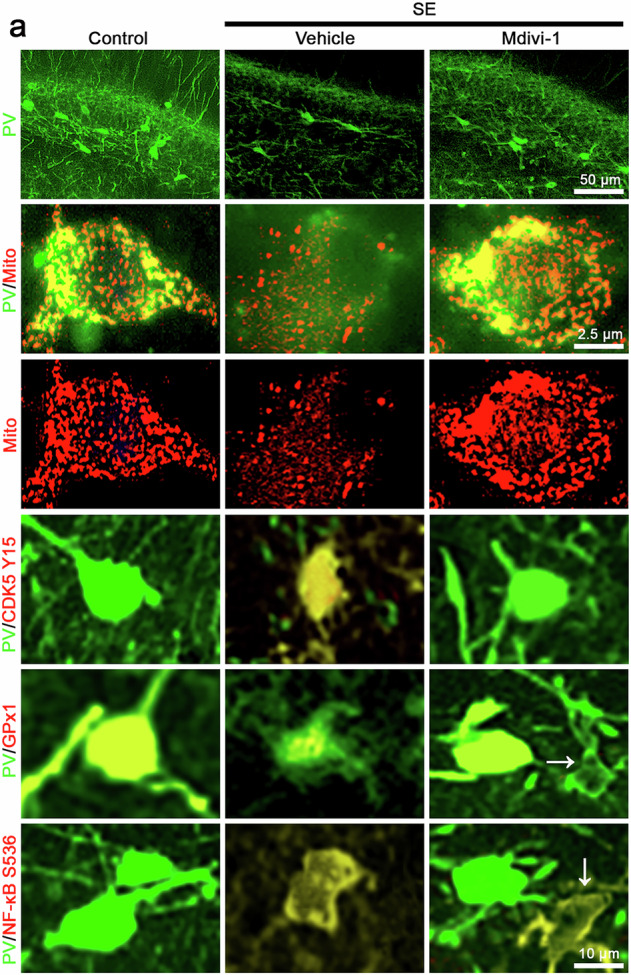


Amended Fig. 6
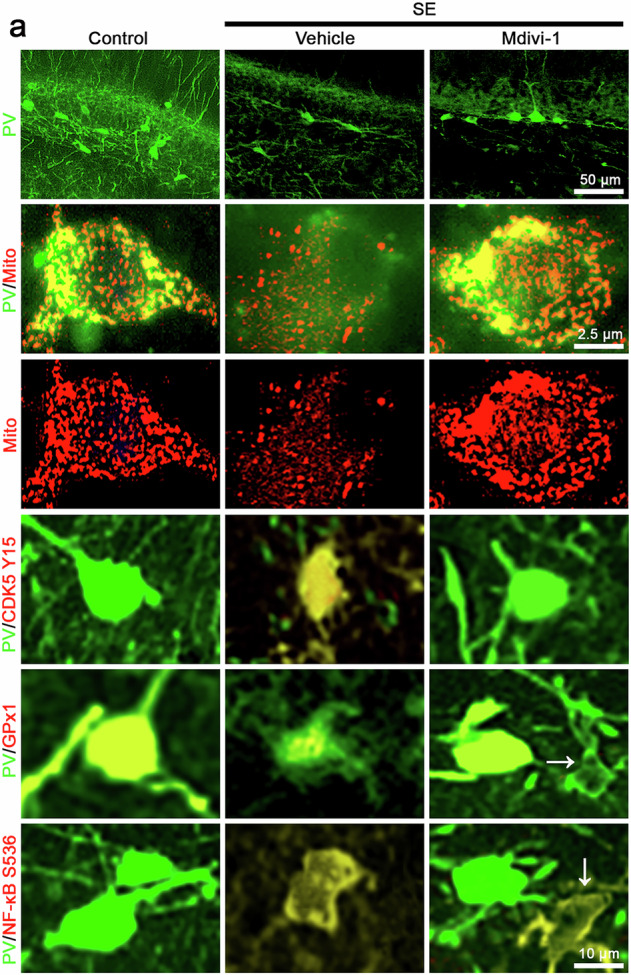


The original article has been corrected.

