# Supplementary material for: Reciprocal regulation of oxidative stress and mitochondrial fission augments parvalbumin downregulation through CDK5-DRP1- and GPx1-NF-κB signaling pathways
Source: Cell Death Dis. 2024 Sep 30;15(9):707. doi: 10.1038/s41419-024-07050-5 (PMC11443148; doi:10.1038/s41419-024-07050-5)
Supplement: Supplementary file 1 — Supplementary information [file 41419_2024_7050_MOESM1_ESM.pdf]

Supplementary Information

Reciprocal regulation of oxidative stress and mitochondrial fission augments parvalbumin downregulation through CDK5-DRP1- and GPx1-NF-κB signaling pathways

Su Hyeon Wang, Duk-Shin Lee, Tae-Hyun Kim, Ji-Eun Kim[*] and Tae-Cheon Kang[*]

Department of Anatomy and Neurobiology, Institute of Epilepsy Research, College of Medicine, Hallym University, Chuncheon 24252, South Korea

* Correspondence to: J.-E. Kim, Department of Anatomy and Neurobiology, College of Medicine, Hallym University, Chuncheon, Kangwon-Do 24252, South Korea; Tel: +82-33-248-2522; E-mail: jieunkim@hallym.ac.kr and T.-C. Kang, Department of Anatomy and Neurobiology, College of Medicine, Hallym University, Chuncheon, Kangwon-Do 24252, South Korea; Tel: +82-33-248-2524; Fax: +82-33-248-2525; E-mail: tckang@hallym.ac.kr

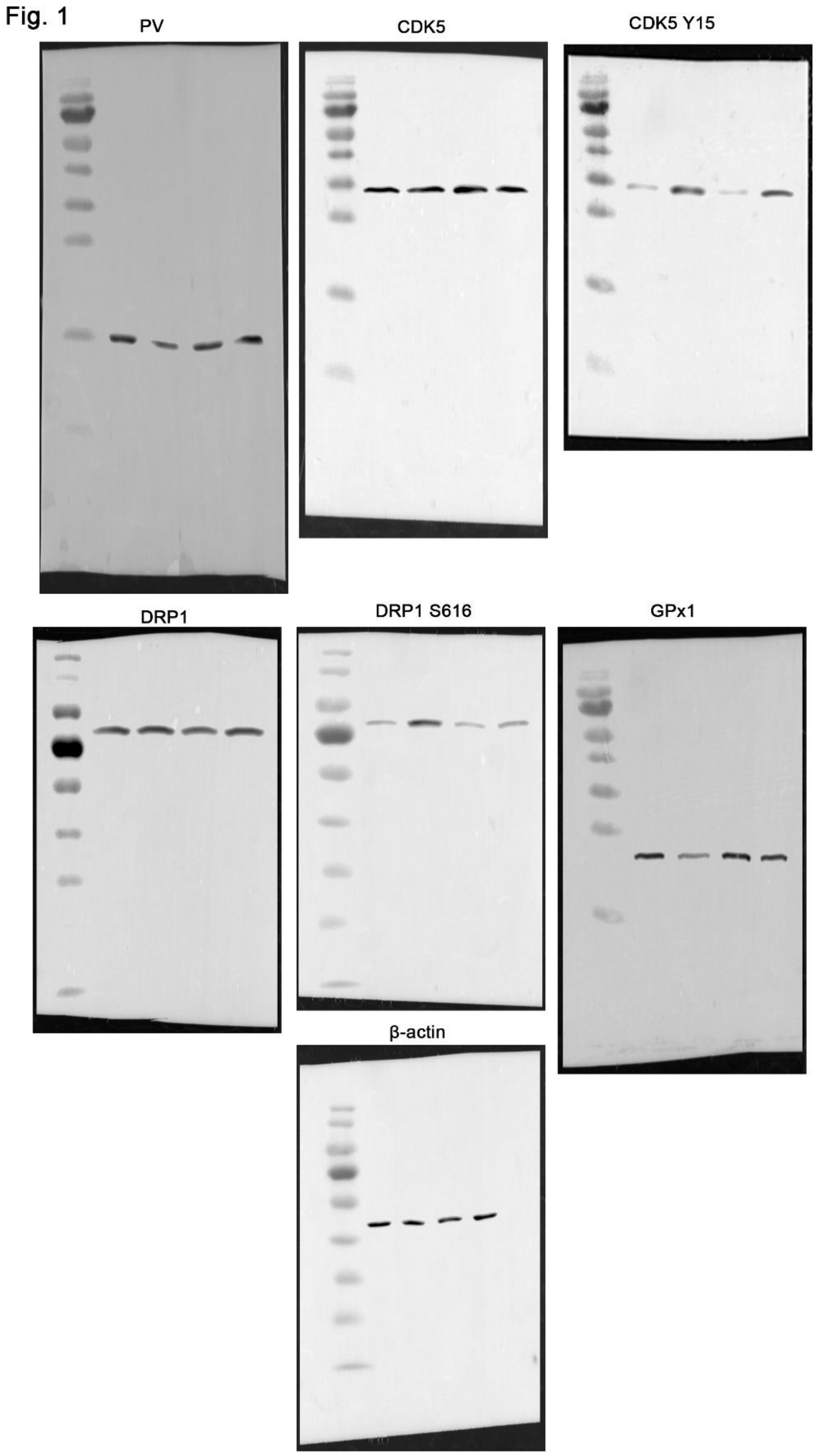

Supplementary Figure 1. Full-gel images of Western blot in Figure 1.
