# Supplementary figures and images for: Reciprocal regulation of oxidative stress and mitochondrial fission augments parvalbumin downregulation through CDK5-DRP1- and GPx1-NF-κB signaling pathways
Source: Cell Death Dis. 2024 Sep 30;15(9):707. doi: 10.1038/s41419-024-07050-5 (PMC11443148; doi:10.1038/s41419-024-07050-5)

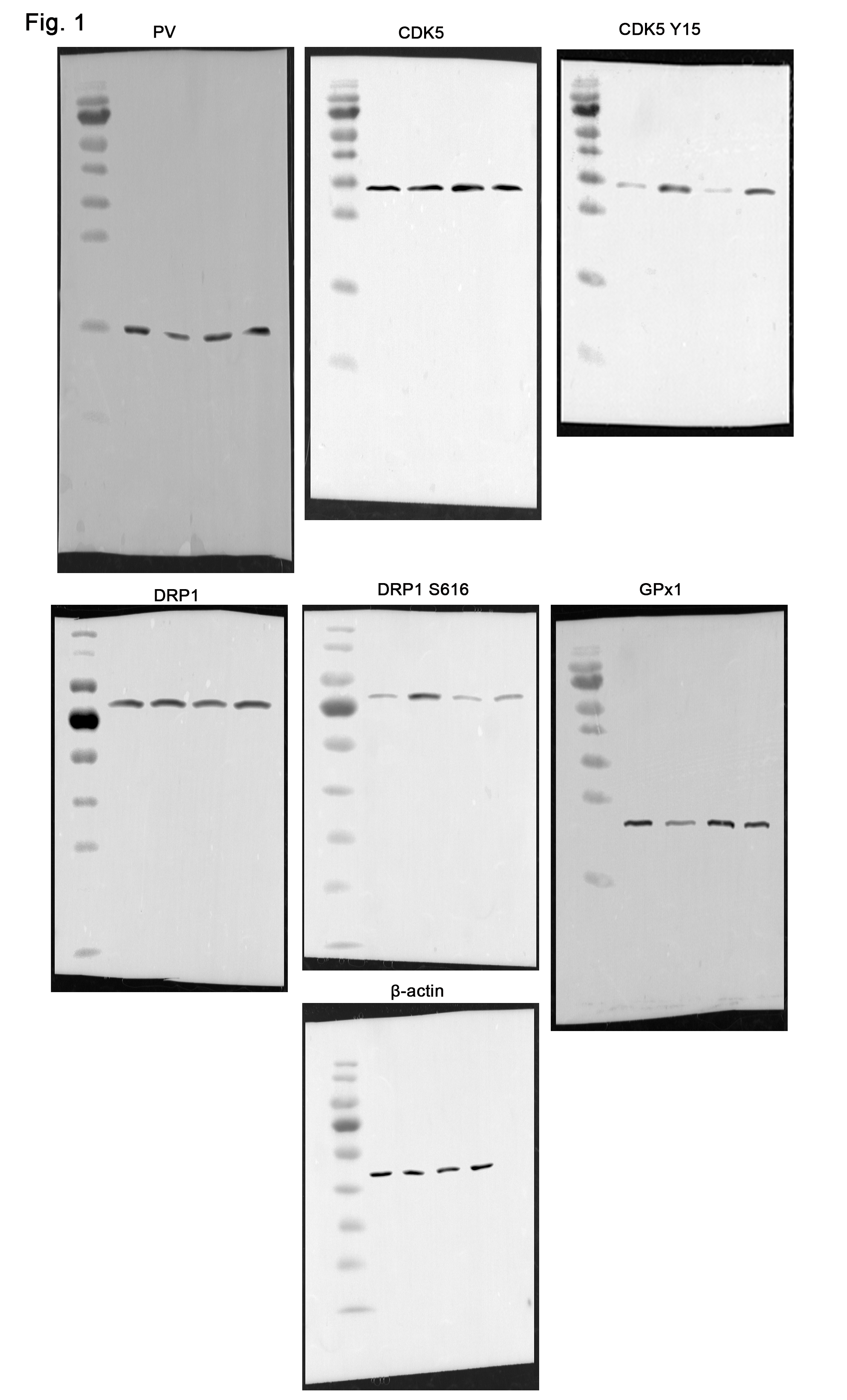

Supplement: Supplementary file 2 — Supplementary Figure 1. Full-gel images of Western blot in Figure 1 [file 41419_2024_7050_MOESM2_ESM.tif]
